# Treatment of Recurrent Hepatocellular Carcinoma with Sorafenib in a HIV/HCV Co-Infected patient in HAART: A Case Report

**DOI:** 10.1186/1750-9378-7-15

**Published:** 2012-06-28

**Authors:** Pasquale De Nardo, Magdalena Viscione, Angela Corpolongo, Rita Bellagamba, Giovanni Vennarecci, Giuseppe Maria Ettorre, Elisa Gentilotti, Chiara Tommasi, Emanuele Nicastri

**Affiliations:** 1 Clinical Department of Infectious Diseases, National Institute for Infectious Diseases IRCCS “L. Spallanzani”, via Portuense 292, 00149, Rome, Italy; 2 General and Transplant Surgery, San Camillo-Forlanini Hospital, via Portuense 292, 00149, Rome, Italy

**Keywords:** HAART, Sorafenib, Fosamprenavir, TDM, Hepatocarcinoma, HIV/HCV co-infection

## Abstract

**Background:**

Liver disease is the second cause of death among HIV patients receiving highly active antiretroviral therapy (HAART) in Europe. HIV patients have a high prevalence of chronic HBV (6–10%) and HCV (33%) co-infection, and accelerated progression of viral hepatitis. Furthermore, the long duration of both HIV and HCV diseases in the HAART era increases the risk of hepatocellular carcinoma.

**Findings:**

We report the case of a 49 year -old HIV/HCV co-infected male patient who developed hepatocellular carcinoma. The patient underwent a partial hepatectomy, and a few months later was treated with transcatheter arterial chemoembolisation due to hepatocarcinoma recurrence. Two months later, advanced hepatocellular carcinoma was diagnosed and sorafenib therapy was initiated. The patient achieved partial response of the main lesions, complete regression of the smallest lesions and did not experience clinical progression during the 20-month follow-up period. During therapy with sorafenib, the patient was treated with HAART with good viral and immunological responses. We used the therapeutic drug monitoring to assess antiretroviral concentrations during co-administration of sorafenib. Fosamprenavir C_trough_ was found under the minimum level recommended by international guidelines. No grade 3 or 4 toxicities were observed. At month 20 of treatment, new liver lesions with portal vein thrombosis were diagnosed. After 28 months of sorafenib therapy, the patient deceased for severe liver insufficiency.

**Conclusions:**

Sorafenib monotherapy demonstrated a marked delay in HCC disease progression in an HIV/HCV co-infected patient. Fosamprenavir C_trough_ was found under the minimum level recommended by international guidelines, suggesting a possible interaction.

## Findings

### Introduction

People infected with HIV have a greater prevalence of chronic HBV (6–10%) and HCV (33%) and accelerated progression of viral hepatitis than the general population [1]. Since the initiation of highly active antiretroviral therapy (HAART) in 1996, the incidence of AIDS-related morbidity and mortality has dramatically decreased, resulting in increased life expectancy. However, the causes of death have shifted from AIDS-defining to non -AIDS defining diseases with increased risk of end-stage liver diseases (ESLD) [2]. According to several studies, the complications of HCV and HBV are the second most frequent cause of death after AIDS in HIV- infected patients, accounting for around 10% of deaths [2,3]. Two large, phase III trials demonstrated that the orally active multikinase inhibitor sorafenib is effective in leading to a longer median overall survival time, and time to progression in patients with advanced hepatocellular carcinoma (HCC) [4,5]. Despite the clinical relevance of HCC in HIV-HCV co-infection, there is little data regarding the use of sorafenib for HCC in HIV/HCV co-infected patients. Furthermore, data on a possible interaction between sorafenib and antiretrovirals are rather scarce [6-8].

### Case report

**Figure 1:**
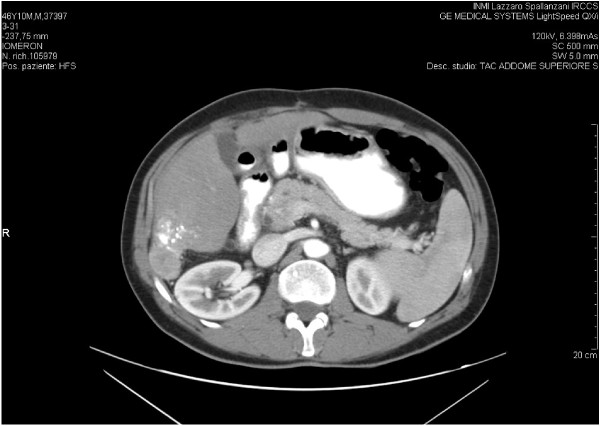
**Abdomen TC scan before starting sorafenib.** Non-homogenic solid nodular lesion on the resection margin of the hepatectomy.

**Figure 2:**
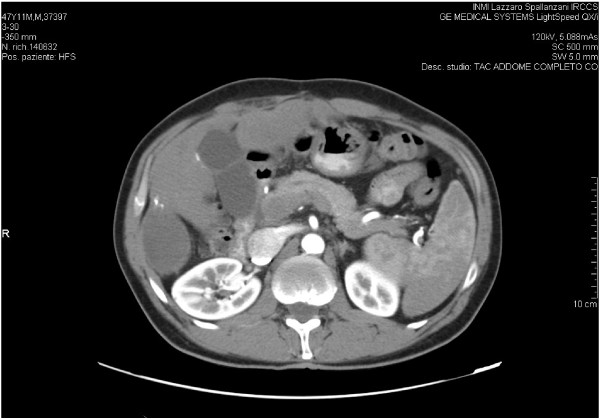
**Abdomen TC scan: follow up during sorafenib treatment.** Right subdiaphragmatic fluid collection with no appearance of the solid nodular lesion (same anatomic level of Figure 1).

A 49 year-old Italian male with HIV infection (known since 1988), and CDC stage C3 for pulmonary tuberculosis, presented an HCV infection (genotype 4) since 1992. His HBV-DNA and hepatitis B surface antigens were negative. He was on antiretroviral therapy since 1991 with good immune-virological control. In December 2005, a liver biopsy showed a moderate grade of necroinflammatory activity and a moderate intensity of fibrosis (Ishak grading 13, Ishak staging 3). HAART was interrupted in March 2006, and in May 2006, he started antiviral treatment with Peg-interferon 180 mcg/week and ribavirin 1000 mg/day. Sixteen weeks later, antiviral therapy was discontinued for virological failure (> 300.000 HCV-RNA UI/mL). In June 2007, 15 months after HAART interruption, the patient started another antiretroviral therapy with emtricitabine (FTC), tenofovir (TDF), fosemprenavir (FPV) and ritonavir (RTV) (100 mg daily): the C_trough_ determination of FPV was 1176 ng/mL, far above the C_trough_ levels suggested by international guidelines (>400 ng/mL) [9]. In July 2007, an abdomen computed tomography scan (TC) showed a single nodule of 4 cm in the VI hepatic segment. A histological diagnosis of HCC was made and he underwent a partial hepatectomy. In April 2008, a new liver lesion of 2.4 cm in the VII-VIII segment was detected and treated with Transcatheter Arterial Chemoembolisation (TACE). Three months later, an abdomen CT scan revealed a right subdiaphragmatic fluid collection and a non-homogenic solid nodular lesion on the resection margin of the previous hepatectomy (Figure 1). Smaller lesions were described in II, III and IV segments. His α-fetoprotein was 20.43 IU/ml. In December 2008, the patient started treatment with sorafenib 800 mg/die, which was reduced to 400 mg/day after 2 months due to a grade II hand-foot skin adverse reaction. Child-Pugh cirrhosis score at the beginning of sorafenib treatment was 6, grade A. The radiological controls during follow-up, until month 20 of therapy showed complete regression of the described nodular lesion with no progression of the smaller lesions (Figure 2). No appearance of new relapses was observed. Fosamprenavir C_trough_ was found under the minimum level recommended by international guidelines (C_trough_: 115 ng/mL; Cmax: 436 ng/mL). Nevertheless, HIV-RNA was still undetectable. At month 20 of treatment, we found an increment of α-fetoprotein up to 200 IU/ml, associated with new hepatic lesions and occurrence of a complete portal vein thrombosis. Sorafenib administration continued after the documented disease progression. In April 2011, after 28 months of sorafenib therapy, the patient deceased for severe liver insufficiency. A summary of CD4 cell count and HIV-RNA values is showed in Table 1. Over the entire period of treatment with sorafenib the patient had taken PI- based HAART, which was well-tolerated, and had maintained a good viral immunological response (CD4 > 500/mmc and undetectable viremia), despite low levels of fosamprenavir concentration. The more severe toxicities correlated to sorafenib assumption were grade II hand-foot skin reaction which required a dose reduction.

**Table 1:** Summary of CD4 cell count and HIV-RNA values

	**CD4 cell/mmc (%)**	**HIV-RNA (cp/mL)**	**Notes**
Mar 2006; May 2006	574 (15.5)	<50	Stop HAART; Start Peg-IFN + RIBA
Sep 2006	357 (18.8)	12596	Stop Peg-IFN + RIBA
Oct 2006 - Sep 2007	>350	≥30000 ≤ 50000	HCC diagnosis and hepatectomy
Jun 2007	277 (13.2)	85462	Restart HAART
Jan 2008	506 (21.1)	<50	
Apr 2008	384 (23.6)	<50	HCC relapse
Dec 2008	526 (23.7)	<50	Start sorafenib
Jan 2009 - Apr 2011	>500	<50	

HAART: highly active antiretroviral treatment; Peg-IFN: pegylated- interferon; RIBA: ribavirin; HCC: hepatocarcinoma.

## Discussion

HCC is the fifth most common cancer in the world and the third most common cause of cancer death. The main risk factors for developing HCC are the presence of cirrhosis and co-infection with HBV and HCV [10].

HIV co-infection worsens the course of viral hepatitis causing faster progression of fibrosis and earlier development of cirrhosis. On the other hand, the introduction of HAART has increased longevity. Consequently, the manifestations of end-stage liver disease, particularly HCC, are more frequently reported [11].

According to current guidelines, treatment of HCC is the same for both patients with and without HIV infection, although the outcome is worse for HIV-positive patients than their HIV-negative counterparts [11]. Until 2007, chemotherapy had not shown survival benefits for these patients because the HCC is generally a chemoresistant tumor [12]. Sorafenib, a new biological drug recently approved in the EU and USA, has substantially changed the treatment and natural course of advanced unresectable HCC. The introduction of this drug has offered a therapeutic opportunity to patients for whom no effective treatment was previously available. Sorafenib is a tyrosine kinase inhibitor directed against several targets (including vascular-endothelial growth factor VEGFR2, platelet-derived growth factor (PDGFR)-b and Raf kinase) that has demonstrated the ability to inhibit tumor proliferation and angiogenesis *in vitro*. Two randomized, double-blind, placebo-controlled, multicentre, phase III trials (the SHARP trial and the Asia-Pacific trial) have proved that monotherapy with oral sorafenib prolonged median overall survival and delayed the median time to progression in patients with advanced HCC [13].

Three cases of HIV-positive patients treated with sorafenib have been described in literature: two patients co-infected with HBV [6,8] and one patient co-infected with HCV [7]. Our case is the second report describing the use of sorafenib for HCC in an HIV-HCV co-infected patient. Our patient’s survival after administration of sorafenib was remarkably prolonged compared with the overall median survival observed in two recent placebo-controlled trials (20 months vs. 10,7 months [4] and 6,5 [5]), with no grade 3–4 toxicity. Since objective responses for HCC patients treated with sorafenib are very rare, this fuels the hypothesis of a possible synergistic effect of sorafenib with HAART. In particular, ritonavir, a protease inhibitor, can inhibit both in vivo and in vitro the PI3/AKT/mTOR pathway and that could provide a molecular biology rationale for explaining, at least in part, the observed objective response. As previously described [7], a 50% dose reduction of sorafenib to 200 mg twice daily was scheduled for safety reasons. Metabolism of sorafenib occurs primarily in the liver, mediated via cytochrome P450 (CYP) 3A4, and concomitant administration with CYP3A4 inducers or inhibitors may modify sorafenib concentrations [13]. Emtricitabine and tenofovir have no interactions with P450 (CYP) 3A4 enzyme , but fosemprenavir (FPV) and ritonavir (RTV) are both P450 CYP3A4 inhibitors and since sorafenib is metabolized through P450 CYP3A4 that could result in an increase of the active dose of sorafenib explaining the favorable outcome for the patient. Consistent with the limited literature data, a good immune-virological response to HAART was maintained throughout the sorafenib therapy, despite low levels of fosamprenavir concentration. Different from the other two cases, at month 20 of treatment with sorafenib, our patient showed an increase in alpha-fetoprotein values. The occurrence of portal vein thrombosis and new lesions clearly identify HCC disease progression.

In conclusion, sorafenib may represent a good therapeutic option also for the treatment of HIV-HCV co-infected patients with advanced HCC who are not candidates for surgery or palliative care. However, longitudinal clinical trials are required in order to confirm both efficacy and safety of sorafenib in co-administration with antiretroviral drugs.

## Competing interests

None; no financial support was received.

## Authors’ contributions

All authors contributed to the manuscript. PDN was involved in drafting the manuscript and reviewing the literature. AC, MV and RB were responsible for the primary management of the patient. CT and EG helped to draft the manuscript. GV and GME provided the figures and helped to draft the manuscript. EN revised the final manuscript and has given the final approval of the version to be published. All authors have read and approved the final manuscript.
